# Using Skin Markers for Spinal Curvature Quantification in Main Thoracic Adolescent Idiopathic Scoliosis: An Explorative Radiographic Study

**DOI:** 10.1371/journal.pone.0135689

**Published:** 2015-08-13

**Authors:** Stefan Schmid, Daniel Studer, Carol-Claudius Hasler, Jacqueline Romkes, William R. Taylor, Reinald Brunner, Silvio Lorenzetti

**Affiliations:** 1 ETH Zurich, Institute for Biomechanics, Zurich, Switzerland; 2 Bern University of Applied Sciences, Health Division, Bern, Switzerland; 3 University of Basel Children’s Hospital, Orthopaedic Department, Basel, Switzerland; 4 University of Basel Children’s Hospital, Laboratory for Movement Analysis, Basel, Switzerland; University of Pennsylvania, UNITED STATES

## Abstract

**Background and Purpose:**

Although the relevance of understanding spinal kinematics during functional activities in patients with complex spinal deformities is undisputed among researchers and clinicians, evidence using skin marker-based motion capture systems is still limited to a handful of studies, mostly conducted on healthy subjects and using non-validated marker configurations. The current study therefore aimed to explore the validity of a previously developed enhanced trunk marker set for the static measurement of spinal curvature angles in patients with main thoracic adolescent idiopathic scoliosis. In addition, the impact of inaccurate marker placement on curvature angle calculation was investigated.

**Methods:**

Ten patients (Cobb angle: 44.4±17.7 degrees) were equipped with radio-opaque markers on selected spinous processes and underwent a standard biplanar radiographic examination. Subsequently, radio-opaque markers were replaced with retro-reflective markers and the patients were measured statically using a Vicon motion capture system. Thoracolumbar / lumbar and thoracic curvature angles in the sagittal and frontal planes were calculated based on the centers of area of the vertebral bodies and radio-opaque markers as well as the three-dimensional position of the retro-reflective markers. To investigate curvature angle estimation accuracy, linear regression analyses among the respective parameters were used. The impact of inaccurate marker placement was explored using linear regression analyses among the radio-opaque marker- and spinous process-derived curvature angles.

**Results and Discussion:**

The results demonstrate that curvatures angles in the sagittal plane can be measured with reasonable accuracy, whereas in the frontal plane, angles were systematically underestimated, mainly due to the positional and structural deformities of the scoliotic vertebrae. Inaccuracy of marker placement had a greater impact on thoracolumbar / lumbar than thoracic curvature angles. It is suggested that spinal curvature measurements are included in marker-based clinical gait analysis protocols in order to enable a deeper understanding of the biomechanical behavior of the healthy and pathological spine in dynamic situations as well as to comprehensively evaluate treatment effects.

## Introduction

Upper-body kinematics during daily activities such as walking are known to be important for movement control [1]. A comprehensive assessment of gait function including within-trunk and spinal movements can therefore be beneficial in specific patient groups. However, evidence of spinal movement during gait, measured using skin marker-based motion capture systems is still limited to a handful of studies, of which the majority was conducted on healthy subjects and without an appropriate evaluation of the spinal curvature [2–12]. Standard trunk marker sets used in clinical gait analysis such as the “Plug-in Gait full body marker set” [13, 14] do not allow the tracking of spinal curvature and are hence not suitable for the quantification of abnormal spinal movement in patients with complex spinal deformities such as adolescent idiopathic scoliosis (AIS) or pathologies that affect spinal movement in the context of passive or active secondary deviations [15].

For these reasons, an enhanced trunk marker set (IfB marker set) was introduced and validated for the assessment of sagittal lumbar and thoracic curvature in healthy adults [16]. Although the validity was reported as low for the measurement of absolute spinal curvature angles, the marker set appeared to be suitable for the reliable assessment of change in sagittal lumbar and thoracic curvature.

An important aspect to consider in AIS is that the spinal deformity is always three-dimensional with the basic components being intervertebral lordosis (sagittal plane), lateral inclination (frontal plane) and axial rotation (transverse plane), i.e. the vertebral bodies rotate toward the convex side and the spinous processes toward the concave side [17, 18]. In addition, AIS patients were shown to have a distinct asymmetrical intravertebral deformity with its maximum being in the apical region of the curve [19]. Taking these factors into consideration, it is reasonable to assume that a superficial tracking of the spinal curvature with markers placed on the spinous processes will underestimate the curvature formed by the vertebral bodies in the frontal plane. Evidence for this assumption was provided by Herzenberg et al. [20], who radiographically demonstrated that the angle derived from the spinous processes significantly underestimated the Cobb-angle. However, it is plausible that this underestimation of curvature is systematic throughout normal functional activities, thereby still allowing the tracking of spinal movement and the dynamic assessment of changes in curvature.

In addition to the malrotation of vertebral bodies, inaccurate marker placement might also contribute to an under- or overestimation of the spinal curvature. Studies showed that the rate of accurately locating the spinous processes by palpation was somewhere between 45% and 83% with a mean distance of inaccurate identification either above or below the targeted level of 19.3±18.6 mm [21–24]. This suggests that the correct identification of the spinous processes is difficult. In addition, all of the mentioned studies have only quantified the accuracy of spinous process identification in the vertical axis. No evidence is available that describes the accuracy of spinous process identification in the horizontal axis, i.e. lateral displacement from the designated location, which is crucial for understanding scoliosis, particularly in dynamic situations.

Using biplanar radiography and marker-based motion capture techniques, the primary aim of the current study was to explore the static validity of skin marker-based measurements of sagittal and frontal plane spinal curvature angles in a group of patients with AIS. In a secondary aim, the accuracy of spinous process identification by palpation and the impact of inaccurate marker placement on curvature angle measurements in the frontal plane were addressed.

## Methods

### Ethics statement

This study was approved by the local ethics committee (Ethikkommission Nordwest- und Zentralschweiz (EKNZ), Ref.-No.: 33/13). All patients as well as their legal guardians provided written informed consent to participate in this study.

### Subjects

A consecutive sample of ten patients with AIS participated in the current study (Table 1). All participants were scheduled for a routine radiographic examination and were therefore not exposed to additional radiation. Inclusion criteria were an age between 10 and 18 years and the diagnosis of an idiopathic scoliosis with a structural (major) main thoracic curve (types 1–3 according to the Lenke classification [25]), whereas exclusion criteria included any other types of scoliosis (e.g. of neurological origin), previous surgical treatments or injuries to the locomotor system which led to persistent deformities in the lower extremities and the trunk.

**Table 1:** Subject demographics and marker selection for the estimation of the curvature angles in the frontal plane.

Subject	Gender	Age [years]	Height [m]	Mass [kg]	Lenke type	Sagittal Curvature		Thoracolumbar/Lumbar Frontal Curvature				Thoracic Frontal Curvature			
						Cobb Angle [degrees]		Convex.	Cobb Angle		Selected Markers	Convex.	Cobb Angle		Selected Markers
						Lumbar	Thoracic		[degrees]	Boundaries			[degrees]	Boundaries	
1	female	12	1.57	38.5	1	45.1	22.1	left	9.2	T12-L4	T11-L4	right	14.0	T6-T11	T5-T11
2	female	14	1.60	50.8	1	59.1	18.9	left	31.0	T12-L4	T11-L4	right	39.9	T5-T12	T5-T11
3	female	16	1.64	58.8	1	65.1	2.9	left	38.5	T12-L4	T11-L4	right	51.1	T6-T12	T5-T11
4	female	14	1.65	55.5	1	33.5	0.4	left	42.2	T12-L4	T11-L4	right	66.9	T6-T12	T5-T11
5	female	16	1.70	53.0	3	82.6	21.1	left	55.6	T10-L3	T9-L3	right	70.9	T4-T9	T3-T9
6	male	16	1.71	58.4	1	36.9	16.9	left	41.1	L1-L5	L1-L5	right	58.1	T8-L1	T7-L1
7	female	15	1.60	57.3	3	33.6	34.3	left	35.2	T11-L3	T11-L3	right	39.5	T7-T11	T5-T11
8	female	15	1.65	54.4	1	48.1	18.9	right	49.0	T8-L2	T7-L2	left	29.6	T2-T7	C7-T7
9	female	14	1.52	40.9	3	33.0	12.9	left	39.2	L1-L4	L1-L4	right	43.5	T6-T12	T5-T11
10	male	16	1.84	85.5	1	36.0	26.2	right	28.0	T8-T12	T5-T11	left	30.9	T2-T7	C7-T7
	8 female2 male	14.8±1.3	1.65±0.1	55.3±12.7		47.3±16.8	17.5±10.1	2 right8 left	36.9±12.6			8 right2 left	44.4±17.7		

The bottom row contains counts and means ± standard deviations.

### Instrumentation

Standard biplanar radiographic examinations (posterior-anterior and lateral images) of the entire spine while standing were performed, including spherical radio-opaque markers (diameter: 5 mm) that were attached to the skin in a configuration described below. Measurements in the motion analysis laboratory were carried out using standard retro-reflective markers (diameter: 9–14 mm) and a 12-camera motion capture system (Vicon, Oxford, UK) at a sampling rate of 300 Hz.

Selected markers from a previously developed trunk marker set (IfB marker set) [26], placed on the spinous processes of C7, T3, T5, T7, T9, T11 as well as L1-L5, were considered.

### Procedures

Spinous processes of interest were located and marked by an experienced physiotherapist using a skin-compatible pen and with the patient in an upright seated position. First, the spinous process of C7 was located using two different methods: palpation of the two most prominent cervical spinous processes and identification of C7 by 1) flexion and assisted extension of the cervical spine (spinous process that remained stationary) [24, 27, 28] and 2) flexion and assisted rotation of the head (greater movement of spinous processes of C6 and C7 than T1) [29]. In a second step, spinous processes were counted down until L5 was identified. Finally, locations were confirmed when the spinous process of L4 corresponded to the level of the imaginary line between the two iliac crests [21, 28] and all identified spinous processes still corresponded to the respective spinal levels in a standing position. Subsequently, radio-opaque markers were placed directly onto the skin and the participants underwent the radiographic examination. After the examination, the radio-opaque markers were replaced with retro-reflective markers and the participants underwent an upright standing static measurement for the period of 2 seconds in the motion analysis laboratory.

### Data analysis/reduction

**Fig 1:**
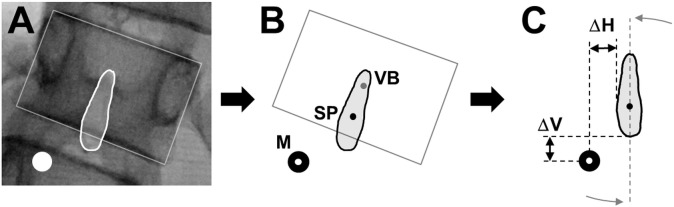
Illustration of the data extraction procedures from the posterior-anterior radiographic images. A) Identification of the shapes of the marker, vertebral body and spinous process, B) calculation of the centers of area (CoA) of the marker (M), vertebral body (VB) and spinous process (SP) and C) calculation of the horizontal (ΔH) and vertical (ΔV) distance between the marker’s CoA and the spinous process boundaries.

Radiographic images were processed using the software ImageJ (version 1.47, U. S. National Institutes of Health, Bethesda, MD, USA). Cobb-angles were determined as described in the literature [30]. The centers of area (CoA) of the vertebral bodies and the markers in both planes as well as the spinous processes in the frontal plane were calculated based on their manually identified shapes (Fig 1A and 1B). Possible errors resulting from the geometry of a diverging x-ray beam were not corrected because of too many unknown variables. The positions of the retro-reflective markers in the sagittal and frontal planes were extracted as the mean values during the 2 seconds static trial using the software Nexus (version 1.8.5, Vicon, OMG, Oxford, UK). All subsequent calculations were carried out using a custom-built MATLAB routine (R2013b, MathWorks Inc., Natick, MA, USA).

Curvature angle calculation in the sagittal and frontal planes was based on the radiographic (vertebral bodies and radio-opaque markers) and motion capture data (retro-reflective markers). In the sagittal plane, the lumbar curve was defined by the vertebral bodies T12-L5 and the markers placed on T11, L1-L5, while the thoracic curve was defined by the vertebral bodies T3-T12 and the markers placed on T3, T5, T7, T9 and T11. In the frontal plane, thoracolumbar / lumbar and thoracic curves were defined by the vertebral bodies and markers that corresponded to the Cobb-angle boundaries (Table 1), with a minimum of four markers selected for each curve. Circular segments were then established using the combination of a second order polynomial and a circle fit function (Taubin method [31]) and curvature angles were calculated based on the central angle theorem.

The accuracy of marker placement by palpation was evaluated on the basis of the posterior-anterior radiographic images (spinous processes and radio-opaque markers). Due to the laterally deviated spine, the calculated CoA’s of the spinous processes and the markers were vertically aligned according to the tilt angle of the longitudinal axis of the respective spinous process (Fig 1C). Vertical accuracy was then determined by measuring the absolute distances (in mm) from a marker’s CoA to the upper and lower boundaries of the respective spinous process and horizontal accuracy by the absolute distances (in mm) from a marker’s CoA to the lateral boundaries of the spinous process. If a marker’s CoA fell between the upper and lower and/or lateral boundaries, the spinous process was considered as correctly identified in the respective direction.

### Statistical analyses

Statistical calculations were performed using the software package SPSS 21 (SPSS Inc., Chicago, IL, USA) and statistical significance was set at p = 0.05.

Primary outcomes: To determine the validity of skin marker-based curvature angle measurements, linear regression analyses among the parameters vertebral body (VB)-, radio-opaque marker (RO)- and retro-reflective marker (RR)-derived curvature angles were carried out.

Secondary outcomes: The accuracy of marker placement by palpation was analyzed descriptively and expressed as numbers and percentages. In addition, absolute displacement values of the markers that were not correctly identified were presented. To explore the impact of inaccurate marker placement on curvature angle measurements in the frontal plane, linear regression analyses among the parameters spinous process (SP)-, RO- and RR-derived curvature angles were carried out with higher correlations indicating a smaller impact and lower correlations a greater impact.

## Results

**Fig 2:**
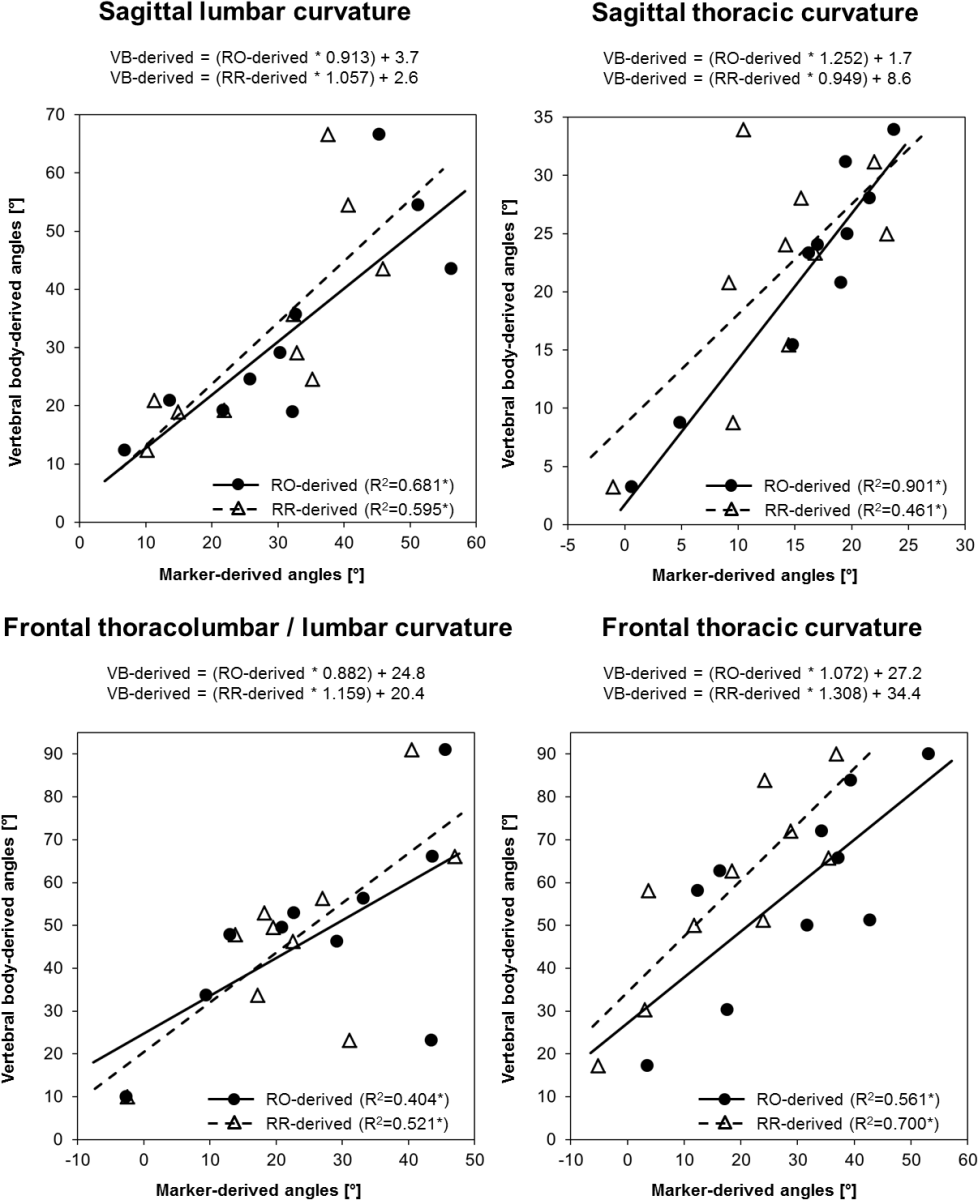
Scatterplots and regression equations illustrating curvature angle estimation accuracy. VB: vertebral body-derived curvature angles; RO: radio-opaque marker-derived curvature angles; RR: retro-reflective marker-derived curvature angles. The asterisks (*) indicate statistical significance at the level p≤0.05.

Primary outcomes: Statistically significant moderate to strong correlations between the VB- and RO- as well as RR-derived curvature angles were found for the sagittal as well as frontal curves (Fig 2). Considering the attributes of the fitted regression lines, the VB-derived curvature angles of the sagittal lumbar and thoracic spines showed no substantial over- or underestimation by the RO and RR markers with slope-values ranging from 0.913 to 1.252 and y-intercept-values of below 10 degrees. In the frontal plane, however, VB-derived curvature angles were systematically underestimated when derived from the markers with slope-values between 0.882 and 1.308 and y-intercept-values of 20.4 to 34.4 degrees. The qualitative consideration of the spread of the sagittal curvature angles in the lumbar spine indicated a more accurate estimation of values less than 40 degrees. In addition, frontal thoracic curvature angles showed a slightly increased underestimation by the RR- compared to the RO-derived values.

**Fig 3:**
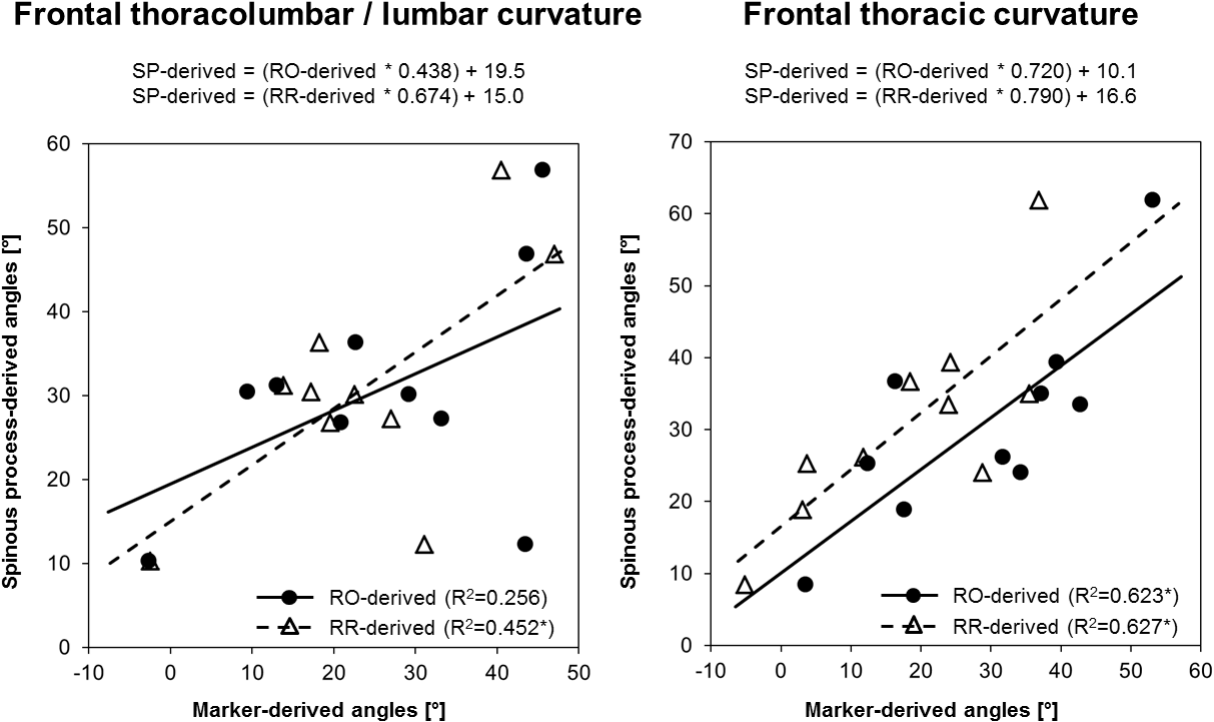
Illustration of the impact of marker placement error on curvature angle estimation. SP: spinous process-derived curvature angles; RO: radio-opaque marker-derived curvature angles; RR: retro-reflective marker-derived curvature angles. The asterisks (*) indicate statistical significance at the level p≤0.05.

Secondary outcomes: Six of the 110 radio-opaque markers were not visible on the radiographs and could therefore not be included in the evaluation. A total of 57.7% of the spinous processes were palpated correctly in the vertical and 38.4% in the horizontal direction (Table 2). Mean displacement values indicated palpatory inaccuracies between 5–18 mm in the vertical and up to 9 mm in the horizontal direction. Spinous processes were generally identified below the designated locations and towards the concave sides of the curves. Regression analyses between the SP- and skin marker-derived curvature angles in the frontal plane showed higher correlations for the thoracic than the thoracolumbar / lumbar spine (Fig 3). In addition, the attributes of the fitted regression lines for the thoracolumbar / lumbar curve indicated a tendency for underestimation of the SP-derived curvature angles, whereas for the thoracic curve, no substantial under- or overestimation could be found.

**Table 2:** Accuracy of spinous process identification by palpation.

		Vert.+Horiz. correct		Only vert. displaced		Only horiz. displaced		Vert.+Horiz. displaced		Missing marker	Displacement* [mm]			
Region	Marker	N	%	N	%	N	%	N	%	N	Vertical		Horizontal	
											Mean	SD	Mean	SD
Cervical	C7	0	0.0	5	100.0	0	0.0	0	0.0	5				
Thoracic	T3	2	22.2	0	0.0	5	55.6	2	22.2	1	5.8	1.2	-1.6	0.8
	T5	3	30.0	2	20.0	3	30.0	2	20.0	0	-6.3	8.1	-3.4	2.3
	T7	0	0.0	2	20.0	6	60.0	2	20.0	0	-6.3	14.1	-8.8	7.2
	T9	0	0.0	1	10.0	6	60.0	3	30.0	0	-7.2	4.7	-4.7	4.7
	T11	1	10.0	2	20.0	4	40.0	3	30.0	0	-17.4	14.2	3.1	2.3
	T3-T11	6	12.2	7	14.3	24	49.0	12	24.5	1	-6.3	8.2	-3.1	4.4
Lumbar	L1	2	20.0	1	10.0	5	50.0	2	20.0	0	-13.3	13.2	0.5	0.2
	L2	2	20.0	1	10.0	4	40.0	3	30.0	0	-11.9	8.8	2.5	1.2
	L3	5	50.0	2	20.0	1	10.0	2	20.0	0	-8.6	3.5	0.6	0.4
	L4	3	30.0	2	20.0	3	30.0	2	20.0	0	-7.3	1.7	1.3	1.0
	L5	2	20.0	2	20.0	3	30.0	3	30.0	0	-11.8	2.1	-0.4	1.7
	L1-L5	14	28.0	8	16.0	16	32.0	12	24.0	0	-10.6	2.5	0.9	1.1
All	C7-L5	20	19.2	20	19.2	40	38.5	24	23.1	6	-8.2	5.9	-1.3	3.5

Numbers and percentages: markers that were placed correctly in both directions and those that were displaced in only the vertical, only the horizontal and both the vertical and horizontal directions. Displacement values: vertical displacement: positive = above designated spinous process, negative = below; horizontal displacement: positive = towards the convex side of upper curve, negative = towards concave side of upper curve.

## Discussion

The current study aimed at validating skin marker-based measurements of spinal curvature angles in the sagittal and frontal planes in patients with main thoracic AIS. In addition, the accuracy of marker placement by spinous process palpation and the impact of inaccurate placement on spinal curvature angle measurements were addressed.

In the sagittal plane, lumbar and thoracic curvature angles could be estimated with a reasonable accuracy by both the RO- and RR-markers, whereas in the frontal plane, thoracolumbar / lumbar and thoracic curvature angles were systematically underestimated by the skin markers. About half of the spinous processes were not identified correctly by palpation in the vertical and another half in the horizontal direction, but with fairly low displacement values. Inaccurate placement of markers appeared to have a greater impact on curvature angle estimation in the thoracolumbar / lumbar than the thoracic spine. While the consequence of these results for assessing the kinematics of the spine during dynamic activities remains to be elucidated in further studies, these data suggest that it might be possible to perform a systematic correction of the marker data or to focus on movement patterns (relative angular differences) in order to provide clinical understanding to dynamic data.

Only few studies evaluated the validity of skin marker-derived spinal curvature measurements [16, 32, 33]. In the late 80’s, Bryant et al. [32] investigated the accuracy of estimating sagittal thoracic and lumbar spine curvatures using RO markers and upright standing lateral view radiographic images in healthy adolescents. Their results suggested accurate estimation of vertebral centroid curves by the RO markers. The thoracic kyphosis was more reliably measured than the lumbar lordosis, possibly due to a greater soft tissue thickness in the lumbar region. Despite the slightly different methodological approach, the current findings on AIS patients largely support these results. Moreover, the fact that a lumbar lordosis of more than approximately 40 degrees was less accurately estimated in AIS patients indicated that the issue of greater soft tissue thickness in the lumbar region seems to also depend upon the extent of the lordotic posture.

To investigate the validity of skin marker-based measurements for the quantification of spinal movement in the sagittal plane, Mörl and Blickhan [34] used MRI images of healthy adults in different seating postures and showed that lumbar vertebral position and spatial orientation could be estimated using skin markers. Using a similar method, Zemp et al. [16] examined soft tissue artifacts as well as estimation accuracies of skin markers (same placement as in the current study) for the measurement of lumbar and thoracic curves. They concluded that skin markers were suitable for the assessment of change in the sagittal curvature angles, but that absolute values suffered from uncertainty. Considering the tendency for lower estimation accuracy with increasing lumbar lordosis as reported in the current study, their suggestions to use skin markers for the assessment of postural change rather than absolute angles should also be followed for measurements in AIS patients.

While the two MRI-based studies above only described spinal curvature measurements in the sagittal plane, Hashemirad et al. [33] validated spinal curvature measurements in the frontal plane using posterior-anterior fluoroscopy images in a neutral and a lateral bending position. Their results suggested that skin markers could be confidently used for the estimation of lumbar spine curvature during lateral bending. Therefore, even though frontal curvatures in the current study were clearly underestimated mainly due to the rotational deformities in AIS patients (i.e. axial rotation and intrinsic axial torsional deformity of the vertebrae), skin markers might still be used for the assessment of movement in this plane.

The current study further showed that VB-derived sagittal thoracic curvature angles could be better estimated by the RO than the RR markers. This might be explained by the fact that in the movement analysis laboratory, the standing position of some patients did not exactly match the posture during the radiographic examination. For the lateral view images, patients were required to hold on to a horizontal bar elevating their arms to the front, which might have caused slight positional differences especially in the upper thoracic spine. If possible, future validations should therefore aim to perform the radiographic and motion capturing measurements simultaneously.

Concerning the differences between RO- and RR-derived measurements, the slightly increased underestimation of the frontal plane curvature angles by the RR compared to the RO markers might be due the fact that the RR markers were mounted on a plastic socket that allowed slight tilting towards the concave side when placed on the paravertebral muscles in the area of the curvature’s apex. In general, regardless of the different types of markers, the underestimation of the curvatures in the frontal plane could be explained mainly by the pathology related structural deformity of the vertebrae as well as a systematic marker placement error towards the concave side of the curvatures. The fact that the palpable parts of the spinous processes were in most cases approximately one level below the position of the respective vertebral body (especially in the lower thoracic spine) might have led to an additional falsification of the actual frontal plane curvature.

Considering the available evidence on the identification accuracy of selected spinous processes, the results of the current study seem to be in agreement for the lumbar but not for the cervical spine. Harlick et al. [21] evaluated the identification accuracy of lumbar spine levels in adults (in the vertical direction), which were identified correctly in 47% of the cases (current study: 60%). The average absolute displacement of the markers that were not identified correctly was 19.3±18.6 mm (current study: 10.6±2.5 mm). Other studies on adult and elderly subjects showed palpatory accuracies of 36–61% for the L5 (current study: 50%) and 55–77% for the C7 spinous process (current study: 0%) [22, 24, 28]. The low accuracy for the identification of C7 in the current study might be explained by the fact that the discrimination between C6 and C7 might have been harder in children and adolescents as compared to adults due to the size of the structures. In addition, five out of six markers that were not visible on the radiographs applied to C7. The placement of the thoracic markers could not be compared to the literature since no studies were available investigating the identification accuracy of thoracic spinous processes.

## Conclusions

Skin marker-based motion capture techniques can be used for the non-invasive assessment of spinal curvature angles in the sagittal and frontal planes in patients with AIS. However, while absolute values in the sagittal plane could be measured with reasonable accuracy, frontal plane angles were systematically underestimated, mainly due to the rotational deformities of the scoliotic vertebrae. Skin markers on the trunk should therefore be used for the assessment of movement and postural change (i.e. during dynamic tasks such as walking) rather than for the measurement of absolute angles.

Inaccuracy of marker placement by palpation had a greater impact on the determination of the thoracolumbar / lumbar than the thoracic curvature angles. In order to keep such inaccuracies minimal, only health care professionals with experience in palpation should place markers.

Based on the current and previous findings, it is suggested that spinal curvature measurements are included in marker-based clinical gait analysis protocols. This would enable a deeper understanding of the behavior of the healthy and pathological spine in dynamic situations and would open up the possibility for a more comprehensive evaluation of treatment effects as movement seems to be detected reasonably well. In addition, the data can be used to drive complex spinal models in order to get an insight into the dynamic loading of the spine during movement.
